# 1167. The Value of Influenza Vaccination in the Older Adult Population. A Stochastic Model Estimation of the benefit of vaccination to prevent the Severe Outcomes in the U.S.

**DOI:** 10.1093/ofid/ofad500.1007

**Published:** 2023-11-27

**Authors:** Stephen I Pelton, Van Nguyen, Joaquin F Mould-Quevedo

**Affiliations:** Boston Medical Center, Boston, Massachusetts; VHN Consulting, Montreal, Quebec, Canada; CSL Seqirus Inc., Summit, New Jersey

## Abstract

**Background:**

Influenza is associated with substantial morbidity and mortality in the United States. Increasing influenza vaccination rates among all persons extends the benefits of immunization programs on reducing illnesses, especially those accrued to the older adult population who has the highest risk of severe outcomes. This research assesses the value of influenza vaccination by estimating the number of severe outcomes such as, respiratory and cardiovascular hospitalizations and deaths averted through immunization in the U.S. older adult population.

**Methods:**

Severe hospitalizations and deaths prevented were estimated through a decision-tree model using a stochastic approach. Model’s incidence rate as well as complications probabilities were calibrated to reflect the reported U.S. CDC data. This analysis compares no vaccination of older adult population against influenza immunization with either a standard-dose quadrivalent vaccine (QIVe) or an enhanced quadrivalent vaccine recommended by ACIP. Absolute vaccine effectiveness and relative vaccine effectiveness of enhanced quadrivalent vaccine vs QIVe for hospitalizations averted were obtained from a targeted literature review. Median value and 95% CI were estimated from a 1000 stochastic simulation.

**Results:**

In comparison to no vaccination, the number of respiratory and cardiovascular hospitalizations prevented with QIVe was 49,323 (19,584-91,620) and 93,950 (41,537-186,224), respectively. In addition, the number of respiratory and cardiovascular deaths prevented with QIVe vs no vaccination was 4,481 (1,517-9,701) and 8,277 (3,124-19,548), respectively. In the case, older adults were vaccinated with an enhanced vaccine, such as aQIV, then the number of respiratory and cardiovascular hospitalizations prevented against no vaccination was 52,428 (21,354-96,922) and 99,948 (44,400-197,284), respectively. Lastly, 4,742 (1,612-10,202) and 8,787 (3,314-20,910) were the estimated deaths prevented due respiratory and cardiovascular hospitalizations, respectively.

**Conclusion:**

Influenza vaccination in older adult population reduces almost by 50% severe complications. The use of an enhanced vaccine such as the adjuvanted formulation increases further the clinical benefits.

**Disclosures:**

**Stephen I. Pelton, MD**, CSL Seqirus Inc.: Advisor/Consultant|CSL Seqirus Inc.: Honoraria **Van Nguyen, PhD**, CSL Seqirus Inc.: Advisor/Consultant|CSL Seqirus Inc.: Advisor/Consultant|CSL Seqirus Inc.: Honoraria **Joaquin F. Mould-Quevedo, PhD**, CSL Seqirus Inc.: Employee|CSL Seqirus Inc.: Employee|CSL Seqirus Inc.: Stocks/Bonds|CSL Seqirus Inc.: Stocks/Bonds

